# Presence of ethanol‐sensitive and ethanol‐insensitive glycine receptors in the ventral tegmental area and prefrontal cortex in mice

**DOI:** 10.1111/bph.15649

**Published:** 2021-09-17

**Authors:** Anibal Araya, Scarlet Gallegos, Rodrigo Viveros, Loreto San Martin, Braulio Muñoz, Robert J. Harvey, Hanns U. Zeilhofer, Luis G. Aguayo

**Affiliations:** ^1^ Department of Physiology Universidad de Concepción Concepción Chile; ^2^ PhD Program in Pharmacology Universidad de Chile Santiago Chile; ^3^ School of Health and Behavioural Sciences University of the Sunshine Coast Sippy Downs Queensland Australia; ^4^ Sunshine Coast Health Institute Birtinya Queensland Australia; ^5^ Institute of Pharmacology and Toxicology University of Zürich and Swiss Federal Institute of Technology (ETH) Zürich Zürich Switzerland; ^6^ Present address: Department of Pharmacology and Toxicology Indiana University School of Medicine Indianapolis Indiana USA

**Keywords:** ethanol, glycine receptors, PFC, reward system, subunit composition, VTA

## Abstract

**Background and Purpose:**

Glycine receptors composed of α1 and β subunits are primarily found in the spinal cord and brainstem and are potentiated by ethanol (10–100 mM). However, much less is known about the presence, composition and ethanol sensitivity of these receptors in higher CNS regions. Here, we examined two regions of the brain reward system, the ventral tegmental area (VTA) and the prefrontal cortex (PFC), to determine their glycine receptor subunit composition and sensitivity to ethanol.

**Experimental Approach:**

We used Western blot, immunohistochemistry and electrophysiological techniques in three different models: wild‐type C57BL/6, glycine receptor subunit α1 knock‐in and glycine receptor subunit α2 knockout mice.

**Key Results:**

Similar levels of α and β receptor subunits were detected in both brain regions, and electrophysiological recordings demonstrated the presence of glycine‐activated currents in both areas. Sensitivity of glycine receptors to glycine was lower in the PFC compared with VTA. Picrotoxin only partly blocked the glycine‐activated current in the PFC and VTA, indicating that both regions express heteromeric αβ receptors. Glycine receptors in VTA neurons, but not in PFC neurons, were potentiated by ethanol.

**Conclusion and Implications:**

Glycine receptors in VTA neurons from WT and α2 KO mice were potentiated by ethanol, but not in neurons from the α1 KI mice, supporting the conclusion that α1 glycine receptors are predominantly expressed in the VTA. By contrast, glycine receptors in PFC neurons were not potentiated in any of the mouse models studied, suggesting the presence of α2/α3/α4, rather than α1 glycine receptor subunits.

## Graphical abstract

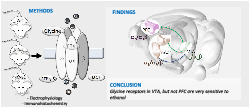

**Abbreviations**
aCSF: artificial CSF
IHC: immunohistochemistry
IR‐DIC: IR differential interference contrast
nAc: nucleus accumbens
NMG: *N*‐methyl‐d‐glucamine
PFC: prefrontal cortex
VTA: ventral tegmental area
α1 KI: glycine receptor α1 knock‐in mice
α2 KO: glycine receptor α2 knockout mice

**What is already known**

- Glycine receptors in the CNS contain α1 and β subunits and are potentiated by ethanol
- There are glycine receptors in higher brain regions, but their exact subunit composition is unknown.

**What does this study add**

- VTA glycine receptors predominantly contain α1 subunits and are highly susceptible to ethanol potentiation.
- Glycine receptors present in the PFC contain α2/α3 subunits and are insensitive to ethanol.

**What is the clinical significance**

- Knowing the glycine receptor composition contributes to understanding the regulation of the reward system.
- Our results may help identify new approaches for the treatment of addiction.

## 1 INTRODUCTION

Ligand‐gated ion channels are an important group of integral membrane proteins responsible for neurotransmission (Kandel et al., 2013; Purves et al., 2004; Rang et al., 2016). Within this group, glycine receptors are the principal inhibitory receptors in the spinal cord and brainstem neurons (Avila, Nguyen, & Rigo, 2013; Burgos et al., 2015; Davies et al., 2003; Kandel et al., 2013). Activation of this type of inhibitory receptor by the amino acid glycine leads to an increase in Cl^−^ flux that effectively reduces the excitability and firing of neurons in circuits controlling sensory transmission, motor control, pain and respiration (Aguayo et al., 1996; Lynch, 2009).

Glycine receptors are composed of α and β subunits, and they can assemble into homopentameric (5α) or heteropentameric (3α/2β or 2α/3β) complexes that localize at synaptic and extrasynaptic sites (Kandel et al., 2013; Lynch, 2009; Purves et al., 2004). Molecular and immunohistochemistry (IHC) studies have described four isoforms of the α subunit and one β subunit, which is widely distributed throughout the CNS (Grenningloh et al., 1990; Malosio et al., 1991; Racca et al., 1998). In neurons of the spinal cord and brainstem, the α2 subunit is switched to α1 during development, which is the most prevalent subunit in these regions in juvenile and adult rodents (Avila, Nguyen, & Rigo, 2013; Jonsson et al., 2012; Malosio et al., 1991). The properties of glycine receptors are dependent on the type of subunit forming the complex. For example, EC_50_ values differ between different glycine receptor isoforms; for example, the α1 subunit is the most sensitive to glycine and the α3 is the least sensitive (Raltschev et al., 2016; Yevenes & Zeilhofer, 2011b). Also, α2 and α3 subunits have splicing variants with different EC_50_ values, and inclusion of β subunits increases their sensitivity to glycine (Miller et al., 2004; Sánchez et al., 2015).

Strychnine, at low concentrations, is a competitive inhibitor of all glycine receptor isoforms. Similarly, picrotoxin blocks all isoforms of glycine receptors, but homomeric receptors are more sensitive than heteromeric receptors (Maleeva et al., 2017; Pribilla et al., 1992). In addition, glycine receptors are affected by several modulators, such as Zn^2+^, Ca^2+^, pH and Cl^−^ (Burgos et al., 2015; Webb & Lynch, 2007), and by general anaesthetics including isofluorane and propofol (Yevenes & Zeilhofer, 2011a). Glycine receptors can also be modulated by the activation of G proteins. For example, the Gβγ dimer has been shown to interact with residues in the large intracellular loop of the α1 glycine receptor subunit, thereby potentiating the glycine‐activated current (Guzman et al., 2009; Yevenes et al., 2003). Ethanol, one of the most important drugs of abuse, also potentiates glycine receptors, and this effect is mediated by Gβγ (Yevenes et al., 2008). This potentiation of glycine receptors by ethanol depends on the subunit composition of the receptor because only α1 and α1β, but not homomeric α2 or α3, are potentiated (Aguayo et al., 2014; Dutertre et al., 2012; Sánchez et al., 2015; Yevenes et al., 2010).

Recent studies have reported the presence of glycine receptors in higher brain regions (Lu & Ye, 2011; McCracken et al., 2017; Salling & Harrison, 2014), where they have been linked to neurological diseases including hyperekplexia (Findlay et al., 2003; Schaefer et al., 2013) and autism spectrum disorder (Pilorge et al., 2016; Zhang et al., 2017), as well as increased alcohol consumption (Blednov et al., 2015; Gallegos et al., 2020; Molander et al., 2005; Muñoz et al., 2019; San Martin et al., 2020). The brain regions involved in reward and drug addiction form a neuronal circuit called the reward system, which includes dopaminergic neurons in the ventral tegmental area (VTA) that innervate the nucleus accumbens (nAc). In addition, the prefrontal cortex (PFC) also receives dopaminergic inputs from the VTA and sends excitatory glutamatergic axons to the nAc (Russo & Nestler, 2013). With the aim of examining the role of glycine receptors in the mesolimbic system, we recently characterized the properties of ethanol‐sensitive glycine receptors in the nAc, finding that although synaptic currents were not affected by ethanol, the strychnine‐sensitive Cl^−^ currents activated by low concentrations of glycine were potentiated by low ethanol concentration (5–10 mM) (Förstera et al., 2017; Gallegos et al., 2019; Muñoz et al., 2018).

Previous studies, primarily in rats, have shown the presence of glycine receptors in the VTA and PFC (Lu & Ye, 2011; McCracken et al., 2017; Salling & Harrison, 2014; Ye, 2000; Zheng & Johnson, 2001; Zhu & Ye, 2005). However, the specific subunit composition and ethanol sensitivity of these receptors have not been determined. To learn about the sensitivity of glycine receptors to glycine and ethanol in VTA and PFC, in addition to WT mice, we studied two genetically modified mouse models: (i) a glycine receptor α1 KI mouse model that has two amino acid mutations in the intracellular loop of the α1 subunit (KK385‐386AA); these mutations reduce GTP‐γ‐S and ethanol potentiation of the glycine receptor. allowing assessment of the functional contribution of the α1 subunit in distinct brain areas (Aguayo et al., 2014; Muñoz et al., 2018); and (ii) a glycine receptor α2 KO mouse model, which enabled us to evaluate the importance of the α2 subunit in these regions (Avila, Vidal, et al., 2013; San Martin et al., 2020).

## 2 METHODS

### 2.1 Animals

All animal care and experimental protocols for this study were approved by the Institutional Animal Care and Use Committee at the University of Concepción. The use of animals complied with Chilean Policies on Humane Care and Use of Laboratory Animals and followed the guidelines for ethical protocols and care of experimental animals established by the National Institutes of Health (NIH, Bethesda, MD, USA). Animal studies are reported in compliance with the ARRIVE guidelines (Percie du Sert et al., 2020) and with the recommendations made by the *British Journal of Pharmacology* (Lilley et al., 2020).

Male and female C57BL/6J (WT), glycine receptor α2‐deficient (α2 KO) and glycine receptor α1 point‐mutated (α1 KI) mice (Aguayo et al., 2014; Avila, Vidal, et al., 2013; Zeilhofer et al., 2005) between 45 and 60 postnatal days were used for the experiments. A breeding colony of male and female C57BL/6J mice was acquired from the Jackson Laboratory (Bar Harbor, ME, USA) (IMSR Cat No. JAX:000664, RRID:IMSR_JAX:000664), and α1 KI and α2 KO mice with a C57BL/6J background were previously described (Aguayo et al., 2014; Avila, Nguyen, & Rigo, 2013; Avila, Vidal, et al., 2013; Muñoz et al., 2018). The knock‐in α1 KK385‐386AA (α1 KI) mice were initially generated in Dr Gregg E. Homanics laboratory (Aguayo et al., 2014) and based on the strategy described by Skvorak et al. (2006) (IMSR Cat No. JAX:023516, RRID:IMSR_JAX:023516). Breeding pairs were transferred from Dr Homanics lab in the United States to Chile where they were bred and maintained in a 12‐h light/dark cycle. GlyT2‐GFP mice (Zeilhofer et al., 2005) (IMSR Cat No. RBRC04708, RRID:IMSR_RBRC04708) were bred and maintained in a 12‐h light/dark cycle in a heterozygous state on a C57BL/6J background (IMSR Cat No. JAX:000664, RRID:IMSR_JAX:000664) allowing the identification of glycinergic innervation. Mice were genotyped as described previously (Zeilhofer et al., 2005). The mouse model lacking the α2 subunit of the glycine receptor (α2 KO mice) was initially generated in the laboratories of Harvey and Dear by deletion of the exon 7 in the *Glra2* gene (Avila, Nguyen, & Rigo, 2013). Breeding pairs were transferred from Dr Rigo's lab in Belgium to Chile where they were bred and maintained in a 12‐h light/dark cycle. α2 KO mice were backcrossed to C57BL/6J (IMSR Cat No. JAX:000664, RRID:IMSR_JAX:000664) and genotyped as described previously (Avila, Nguyen, & Rigo, 2013). All the animals used in this study were generated from crosses between hemizygous males (*Glra2*−/*Y*) and heterozygous females (*Glra2*−/+). Mice were housed in groups of 2–4 in a 12‐h light/dark cycle and given food and water ad libitum. When possible, tissues from each animal were used for multiple experiments.

### 2.2 Experimental protocols

All the studies were designed to generate groups of equal size and randomly assigned. Operators and data analyses were blinded. The group sizes were selected on the basis of the results of previous studies (San Martin et al., 2020).

### 2.3 Western blot

Tissue homogenates after detergent treatment (10 mM Tris–HCl pH 7.4, 0.25 M sucrose, 10 mM NEM and protease inhibitor cocktail 1×) were analysed by electrophoresis on 10% SDS‐PAGE gels. Proteins were blotted onto a nitrocellulose membrane (Bio‐Rad) and blocked with 5% skimmed milk in 1× TBS–0.1% Tween 20 for 1 h with stirring. Subsequently, the membranes were incubated with the following primary antibodies: anti‐glycine receptor pan α (1:500; rabbit monoclonal IgG; Cat No. 146008; Synaptic Systems, Germany; RRID:AB_2636914), anti‐glycine receptor β (1:200, rabbit polyclonal IgG, Cat No. AGR‐014, Alomone, RRID:AB_2340973) and anti‐Gβ (1:600, rabbit polyclonal IgG, Cat No. Sc‐378, Santa Cruz Biotechnology, RRID:AB_631542) for 1–2 h. After washes with 1× TBS and 0.1% Tween 20, membranes were incubated for 1 h with anti‐rabbit secondary antibodies conjugated to HRP (1:3000, goat polyclonal anti‐rabbit IgG‐HRP, Cat No. sc‐2004, Santa Cruz Biotechnology, RRID:AB_631746). The immunoreactivity of the proteins was detected using an ECL Plus Western Blotting Detection System (PerkinElmer, MA, USA). Levels of Gβ were used as loading controls. Western blot was quantified using the ImageJ (NIH) program. The Immuno‐related procedures used comply with the recommendations made by the *British Journal of Pharmacology* (Alexander et al., 2018). The data were expressed in relative units (RU) of the normalization of the signal between glycine receptor pan α divided by Gβ signal (GlyR pan α/Gβ [RU]).

### 2.4 Immunohistochemistry

C57BL/6J and GlyT2‐GFP adult mice were deeply anaesthetized and fixed by vascular perfusion with 4% paraformaldehyde (PFA). Brains were removed and postfixed overnight with PFA at 4°C. Brain slices containing VTA or PFC (40 μm) were obtained on a vibratome (Leica VT 1200S). After three washes with 1× PBS, tissue was blocked with normal horse serum (10%) for 45 min. Slices were incubated (overnight) with a combination of the following primary antibodies: anti‐GFP (1:300, polyclonal rabbit antiserum, Cat No. 132002, Synaptic Systems, RRID:AB_887725) and glycine receptor α1 (1:100, monoclonal mouse purified IgG, Cat No. 146111, Synaptic Systems, RRID:AB_887723), MAP2 (1:200, monoclonal mouse purified IgG, Cat No. 188011, Synaptic Systems, RRID:AB_2147096) or TH (1:400, polyclonal guinea pig antiserum, Cat No. 213104, Synaptic Systems, RRID:AB_2619897). Subsequently, slices were washed with 1× PBS and incubated with a secondary IgG antibody: anti‐rabbit Alexa Fluor 488 (1:200, polyclonal donkey anti‐rabbit, Cat No. 711‐546‐152, Jackson ImmunoResearch, RRID:AB_2340619), anti‐mouse Cy3 (1:200, polyclonal donkey anti‐mouse, Cat No. 715‐165‐150, Jackson ImmunoResearch, RRID:AB_2340813) and anti‐guinea pig Cy3 (1:200, polyclonal donkey anti‐guinea pig, Cat No. 706‐165‐148, Jackson ImmunoResearch, RRID:AB_2340460) diluted 1:200 for 2 h. After five washes with 1× PBS, the preparations were mounted with Dako mounting medium (DakoCytomation, USA). Samples were photographed using confocal microscopy (Zeiss LSM700, Germany). Triple‐colour immunofluorescent images were captured, stored and analysed using the ImageJ program (NIH) (RRID:SCR_003070). IHC was performed according to *BJP* guidelines (Alexander et al., 2018).

### 2.5 Electrophysiology

#### 2.5.1 Preparation of brain slices

Coronal slices containing VTA and PFC were prepared as reported (Jun et al., 2011). After excision, the brain was placed in ice‐cold cutting solution (in mM: sucrose 194, NaCl 30, KCl 4.5, MgCl_2_ 1, NaHCO_3_ 26, NaH_2_PO_4_ 1.2 and glucose 10, saturated with 95% O_2_ and 5% CO_2_ and adjusted to pH 7.4). The brain was glued with the cut surface to the chilled stage of a VT1200S vibratome (Leica, Wetzlar, Germany) and sliced to a thickness of 300 μm. Slices were transferred to artificial CSF (aCSF) solution (in mM: NaCl 124, KCl 4.5, MgCl_2_ 1, NaHCO_3_ 26, NaH_2_PO_4_ 1.2, glucose 10 and CaCl_2_ 2, saturated with 95% O_2_ and 5% CO_2_ at 32°C and adjusted to pH 7.4 and 310–320 mOsm·L^−1^). Brain slices were allowed to rest in O_2_‐perfused aCSF at 32°C for at least 1 h before electrophysiological recording or enzymatic treatment for dissociation.

#### 2.5.2 Preparation of acutely dissociated neurons

Acutely dissociated neurons were prepared from brain slices, and the region of interest was microdissected (Figure S1 shows brain region demarcation). Brain slices containing the VTA and PFC were incubated for 30 min with 0.5‐mg·ml^−1^ pronase (Calbiochem/EDM Bioscience, Darmstadt, Germany) in oxygenated aCSF (95% O_2_/5% CO_2_) at 37°C. VTA and PFC were microdissected from the slices, and the neurons were dissociated by mild mechanical trituration (10 times each with 1000‐ and 200‐μl micropipettes and with a fire‐polished self‐drawn glass pipette) in trituration buffer (in mM: NaCl 20, *N*‐methyl‐d‐glucamine [NMG] 130, KCl 2.5, MgCl_2_ 1, HEPES 10 and glucose 10, adjusted to pH 7.4 and 340 mOsm·L^−1^) and allowed to settle for 15–20 min before recordings. The dissociated neurons are identified by their morphology, size around 10–20 μm, phase contrast birefringence and membrane properties. More importantly, these cells generate action potentials when depolarized (Fernández‐Pérez et al., 2020).

#### 2.5.3 Brain slice recordings

For electrophysiological slice recordings, acute brain slices were transferred to the recording chamber with aCSF solution saturated with 95% O_2_ and 5% CO_2_ at 32°C. The slices were observed on an IR differential interference contrast (IR‐DIC) microscope using 10× and 40× objectives (Nikon Eclipse FN1, Tokyo, Japan) and perfused with oxygenated aCSF at 2 ml·min^−1^ at 30–32°C. Whole‐cell current recordings were performed using the voltage‐clamp technique. Patch pipettes with a resistance of 4–8 MΩ were prepared from filament‐containing borosilicate micropipettes (World Precision Instruments, Sarasota, FL, USA) using a P‐1000 micropipette puller (Sutter Instruments, Novato, CA, USA) and filled with internal solution (in mM: 120 KCl, 4.0 MgCl_2_, 10 BAPTA, 0.5 Na_2_‐GTP and 2.0 Na_2_‐ATP, adjusted to pH 7.4 and 290–310 mOsm·L^−1^). Signals were captured using an Axopatch 200B amplifier (Axon Instruments, Union City, CA, USA) at a holding potential of −60 mV, displayed and stored on a personal computer using a 1322A Digidata device (Axon Instruments). For synaptic experiments, we used receptor antagonists for NMDA ( d‐AP5 40 μM), AMPA (CNQX: 6‐cyano‐7‐nitroquinoxaline‐2,3‐diona 10 μM), GABA_A_ (bicuculline 10 μM) and nicotinic (mecamylamine 10 μM) receptors along with TTX (500 nM) to isolate glycinergic inhibitory postsynaptic currents (mIPSC). Strychnine (4 μM) was used to block glycine receptors. Currents were analysed with Clampfit 10.1 (Axon Instruments, Union City, CA, USA) and MiniAnalysis 6.0 (Synaptosoft Inc.). We show the analysis of frequency (Hz), decay constant (ms) and amplitude (pA). The decay constant of mPSCs was fitted as a single exponential, and both rise and decay phases were fitted between 10% and 90% of the maximal amplitude.

#### 2.5.4 Recordings in dissociated neurons

Dissociated neurons from PFC and VTA were recorded in the voltage‐clamp configuration at −60 mV and at room temperature. The glycine‐evoked current was recorded using an internal solution containing (in mM): 120 KCl, 4.0 MgCl_2_, 10 BAPTA, 0.5 Na‐GTP and 2.0 Mg‐ATP (pH 7.4, 290–310 mOsm). The external solution contained (in mM) 150 NaCl, 2.5 KCl, 2.5 CaCl_2_, 1.0 MgCl_2_, 10 glucose and 10 HEPES (pH 7.4, 315–320 mOsm). The amplitude of the glycine current was measured by using a short pulse (1–2 s) of different concentrations of glycine (1–1000 μM) to obtain a concentration–response curve. The Hill equation: *I*_gly_ = *I*_max_ (gly)^nh^∕[(gly)^nh^ + (EC_50_)^nh^] was used to plot the curves (Wittekindt et al., 2001). The data were normalized against the saturating glycine current of 1000 μM (normalized current [%]). We used an array of external tubes (internal diameter, 200 μm) placed within 50 μm of the neuron and solutions containing the ligands flowed continuously from the tubes by gravity. To determine ethanol potentiation, we analysed the evoked currents, in the presence or absence of 10 to 100 mM ethanol, using a concentration of glycine equivalent to EC_10–20_. This value was estimated from the concentration–response curve and considering a concentration that activated a Cl^−^ current that was stable over time and had an amplitude above the noise level to increase the sensitivity of the measurement. The EC_10–20_ values were 7 and 30 μM glycine for VTA and PFC, respectively, for both WT and α1 KI mice. The EC_10–20_ values used in the study were 15 and 40 μM glycine for VTA and PFC, respectively, for α2 KO mice. The data were expressed as percentage of current changed in the presence of ethanol (ethanol potentiation [%]). The picrotoxin assay was performed according to the protocol of Maleeva et al. (2017), using 30 and 50 μM glycine for VTA and PFC, respectively, and 20 μM of picrotoxin at a holding potential of −30 mV. The data are shown as the chloride‐current inhibition percentage (inhibition [%]). The experiments with GTP‐γ‐S were performed in the presence of the non‐hydrolysable GTP analogue (GTP‐γ‐S) in the internal solution. We analysed its effect on the amplitude of the glycine‐activated currents every 3 min, up until 15 min of dialysis. The data are shown as the percentage of chloride current potentiated by GTP‐γ‐S (potentiation [%]).

### 2.6 Data and statistical analysis

The data and statistical analysis comply with the recommendations of the British Journal of Pharmacology on experimental design and analysis in pharmacology (Curtis et al., 2018). Statistical analyses were performed for studies where each group size was at least *n* = 5, using the two‐tailed paired or unpaired Student's *t* tests, and for non‐normally distributed data, Mann–Whitney *U*‐test was used. We include data not subjected to statistical analysis due to the small group size of *n* < 5 because of the lack of a response to glycine in PFC neurons from the KO α2 mouse. Data with more than two groups or factors were analysed by one‐way or two‐way ANOVA test using Origin 8 (Microcal, Inc., MA, USA) or GraphPad Prism 6 Software (RRID:SCR_002798). After ANOVA, Bonferroni post hoc test was run only if *F* achieved the necessary level of statistical significance (*P* < 0.05) and there was no significant variance in homogeneity. Data are shown as mean ± SEM for normally distributed populations and as median and interquartile ranges (IQRs) for non‐normally distributed populations. The group size in this study represents independent values, and the statistical analysis was done using these independent values. As in previous studies (Aguayo et al., 2014; Muñoz et al., 2019), in order to obtain statistical power above 95% (*α* = 0.05 and power = 0.95) to determine existence of statistically significant differences (*P* < 0.05), we used a sample size of 6–8 measurements for experimental group. Outliers were excluded from the statistical analysis. To identify outliers, we performed the ROUT method and the *Q* was set to 1%.

### 2.7 Materials

Bicuculline and CNQX were purchased from Tocris (Bristol, UK). Glycine and strychnine were obtained from Sigma‐Aldrich (St. Louis, MO, USA). TTX was purchased from Alomone Labs (Jerusalem, Israel). Ethanol was purchased from Merck Millipore (Burlington, MA, USA).

### 2.8 Nomenclature of targets and ligands

Key protein targets and ligands in this article are hyperlinked to corresponding entries in in the IUPHAR/BPS Guide to PHARMACOLOGY (http://www.guidetopharmacology.org) and are permanently archived in the Concise Guide to PHARMACOLOGY 2019/20 (Alexander, Kelly et al., 2019; Alexander, Mathie et al., 2019).

## 3 RESULTS

### 3.1 Presence of glycine receptor α subunits and glycinergic markers in the PFC and VTA

**FIGURE 1:**
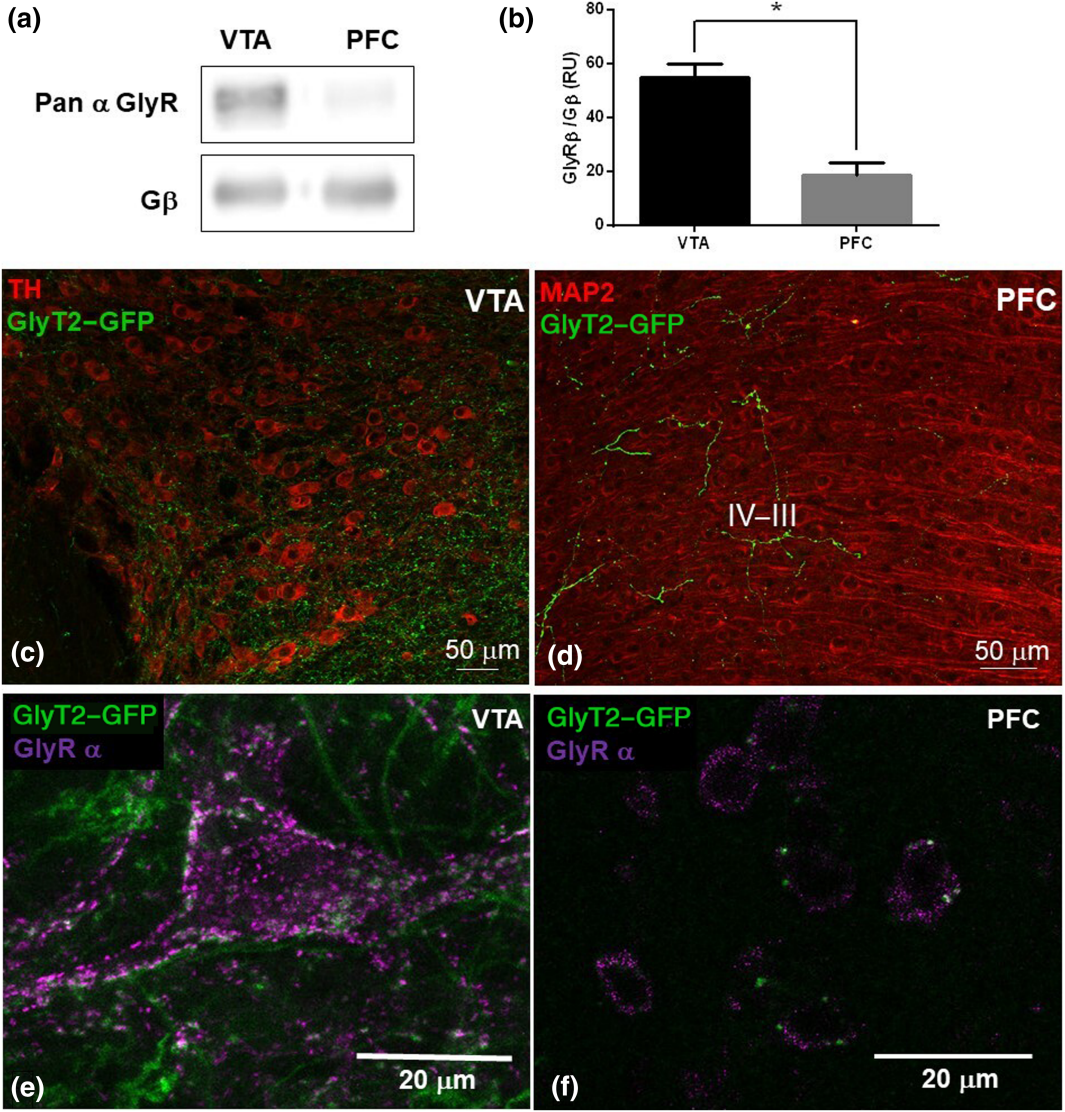
Presence of glycine receptors in the mesolimbic circuit. (a) Western blot shows the presence of the α subunits (MW 48 kDa) of glycine receptors (α GlyR) in the VTA and PFC. (b) Graph represents the normalized signal of α GlyRs to Gβ. Data shown are means ± SEM; *n* = 5 (from duplicates). ^*^ *P* < 0.05, significantly different as indicated; unpaired Student's *t*‐test. (c, d) Low magnification image of VTA and PFC (25×), respectively, in coronal brain slices from a GlyT2‐GFP mouse with TH (red) and anti‐GFP (green) signals in the VTA and MAP2 (red) and anti‐GFP (green) signals in the PFC. GlyT2‐positive projections appear in both regions. (e, f) High magnification image of the VTA and PFC (63×), respectively, in brain slices of a GlyT2‐GFP mouse showing GlyT2‐GFP (green) and GlyR α (magenta) signals. Confocal images of the VTA and PFC were replicated in three different mice

To study the presence of glycine receptor α subunits in these two regions, we performed Western blot analysis using an antibody that recognizes all glycine receptor α subunits and compared their expression to Gβ, a membrane protein (San Martin et al., 2020; Wang et al., 2002) (Figure 1a,b). We found that glycine receptors, normalized to Gβ levels, were expressed in the PFC and VTA, with a statistically significant higher expression in the VTA. To confirm the presence of glycinergic innervation, previous studies have used the presence of GlyT2 protein, which is a neuronal transporter for glycine and a marker of glycinergic axon terminals (Zeilhofer et al., 2005). IHC in GlyT2‐GFP mice showed that the PFC and VTA contain glycinergic axons with different densities. Figure 1c, for instance, shows abundant GFP‐positive fibres in the VTA, with few near to TH‐positive neuronal cell bodies. In the PFC, however, GFP‐positive fibres were more frequently associated with the deeper layers (Figure 1d). Thus, these results suggest the presence of glycinergic synaptic contacts in both regions of the mesolimbic system. We then analysed the presence of different glycine receptor subunits in these regions. IHC using a pan α subunit antibody in brain slices of GlyT2‐GFP mice demonstrated the presence of glycine receptor α subunits in the VTA (Figure 1e) and PFC (Figure 1f). The signal for glycine receptors was found in neuron soma and dendrites. An apposition of glycine receptor immunoreactivity with GlyT2‐GFP signals found in several neurons suggests the presence of synaptic receptors (observed as a white mark with the green and magenta apposition).

### 3.2 Neurons in the VTA and PFC have strychnine‐sensitive glycinergic synaptic currents

**FIGURE 2:**
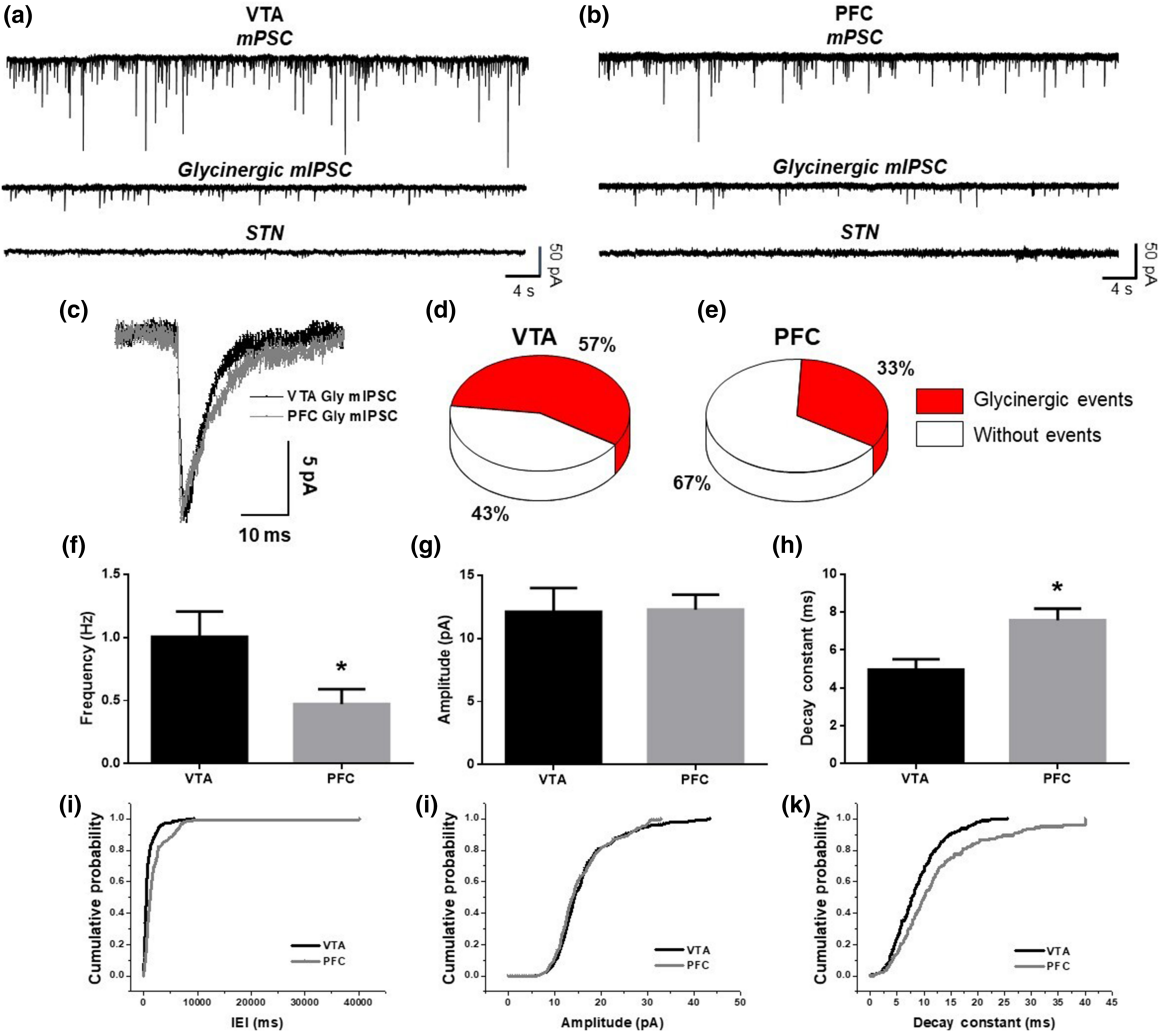
Presence of glycinergic synaptic activity in VTA and PFC in WT mice. (a, b) Representative traces of 1 min of synaptic activity in the VTA and PFC, respectively. The first trace shows the total mPSC, the second shows the isolated mIPSC mediated by glycine receptors and the third trace shows the blockade of the glycinergic mIPSC by strychnine (STN). (c) Average glycinergic mIPSC in the VTA and PFC. (d, e) Percentage of neurons recorded with and without glycinergic activity in the VTA and PFC, respectively. Graph shows the average frequency (f), amplitude (g) and the average decay constant (10–90%) (h) of the glycinergic currents in the VTA and PFC. Graph shows the average cumulative probability for interevent interval (i), amplitude (j) and decay constant (k). Data presented as means ± SEM; *n* = 8 neurons for VTA and *n* = 15 neurons for PFC. ^*^ *P* < 0.05, significantly different from VTA; unpaired Student's *t* test for (f)–(h), and Kolmogorov–Smirnov test for (i)–(k) where significance was achieved for all three parameters

The expression of glycine receptor α subunits demonstrated in our Western blot studies and the presence of GlyT2‐positive presynaptic terminals in IHC suggested the presence of synaptic receptors in these two regions of the reward system. To substantiate the presence of a functional glycinergic innervation, we performed whole‐cell recordings in brain slices from WT mice to evaluate miniature postsynaptic currents (mPSC) in neurons of the VTA and PFC. To isolate glycinergic inhibitory postsynaptic currents (mIPSC), we used a mixture of receptor blockers such as bicuculline (for GABA_A_ receptors), CNQX (for AMPA receptors), d‐AP5 (for NMDA receptors), mecamylamine (for nicotinic ACh receptors) and TTX. The recordings showed the presence of mIPSC in the VTA (Figure 2a) and PFC (Figure 2b) that were blocked by 4 μM strychnine. We detected a higher number of neurons with glycinergic activity in the VTA (57%) compared with the PFC (33%). The mIPSCs found in both brain regions had similar properties as those found in the nAc (Muñoz et al., 2018). The amplitude of the currents was approximately 12 pA in both regions (Figure 2g), but the decay time constants differed between the VTA and the PFC (Figure 2h). The data also showed that the frequency of the glycinergic events was higher in the VTA than in the PFC (Figure 2f), suggesting that there is a higher level of inhibition mediated by glycine receptors in the VTA. The average cumulative probability graphs also showed that the properties of the glycine mIPSCs in PFC and VTA were significantly different (Figure 2i–k).

### 3.3 Distinct properties of glycine‐activated currents in VTA and PFC neurons

**FIGURE 3:**
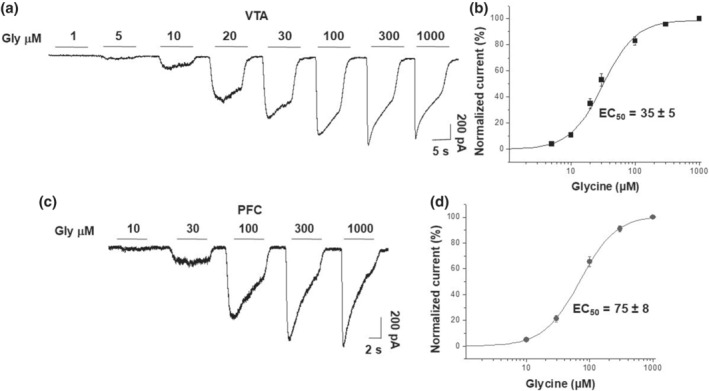
Glycine‐activated currents in dissociated neurons from VTA and PFC in WT mice. (a) Representative traces of currents activated with 1 to 1000 μM glycine in neurons from the VTA. (b) Glycine concentration–response curve normalized to the maximum response. (c) Representative traces of currents activated with 10 to 1000 μM glycine in neurons from the PFC. (d) Glycine concentration–response curve normalized to the maximum response. Data presented are means ± SEM. *n* = 15 from three mice for VTA and *n* = 11 from six mice for PFC. The curves were significantly different (*P* < 0.05); Student's *t* test

We analysed the sensitivity of the glycine receptors in the VTA and PFC to exogenously applied glycine to further characterize the glycinergic currents in the VTA and PFC. The data showed that VTA and PFC neurons had large‐amplitude glycine‐activated Cl^−^ currents and that all neurons recorded in the VTA and PFC responded to glycine, but the glycine receptors in the VTA were more sensitive to glycine. For example, we found that the VTA had currents that were almost 100 pA with 10 μM glycine (Figure 3a). The concentration–response curve to glycine in the VTA (Figure 3b) was shifted to the left as compared with that in the PFC (Figure 3d), with significantly different EC_50_ values. In both cases, the currents were completely and reversibly blocked by strychnine. In agreement with previous results, these data suggest that functional glycine receptors are expressed in these regions and that these glycine receptors have different subunit compositions.

### 3.4 Sensitivity to picrotoxin supports the presence of heteromeric glycine receptors in VTA and PFC neurons

**FIGURE 4:**
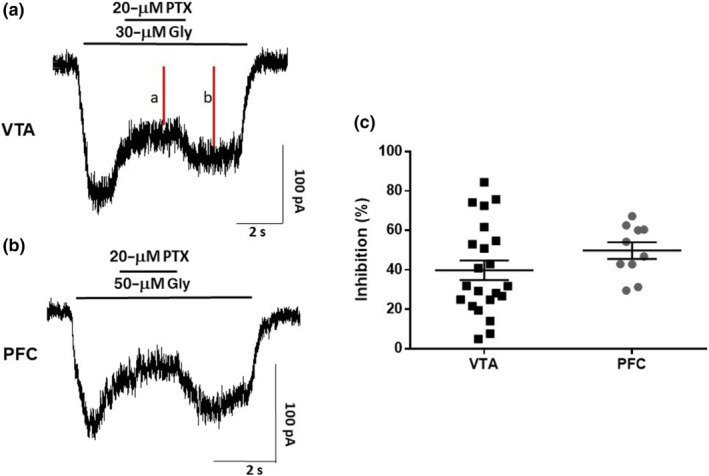
Inhibition of glycine receptors in VTA and PFC neurons by picrotoxin (PTX) in WT mice. (a, b) The blocking action of PTX (20 μM) on glycine currents mediated in neurons from the VTA and PFC, respectively. The traces were evoked with 30 μM glycine for VTA and 50 μM for PFC. (c) Graph shows the percentage of inhibition of the current from control after application of PTX for VTA and PFC. Data presented are means ± SEM. *n* = 22 for VTA and *n* = 10 for PFC. % inhibition = 100 − (*a* × 100∕*b*)

To confirm the presence of functional heteromeric glycine receptors, we performed patch‐clamp recordings in dissociated neurons using picrotoxin because it was reported that this toxin partly blocks heteromeric αβ glycine receptors but it can block homomeric α glycine receptors to a higher extent (Lynch, 2009; Maleeva et al., 2017). The results in Figure 4 showed that application of 20 μM picrotoxin in the VTA and in the PFC clearly inhibited the amplitude of the glycinergic currents to about the same extent, suggesting the presence of heteromeric receptors in both the VTA and the PFC.

### 3.5 Potentiation of glycine receptors by Gβγ in VTA and PFC neurons in WT mice

**FIGURE 5:**
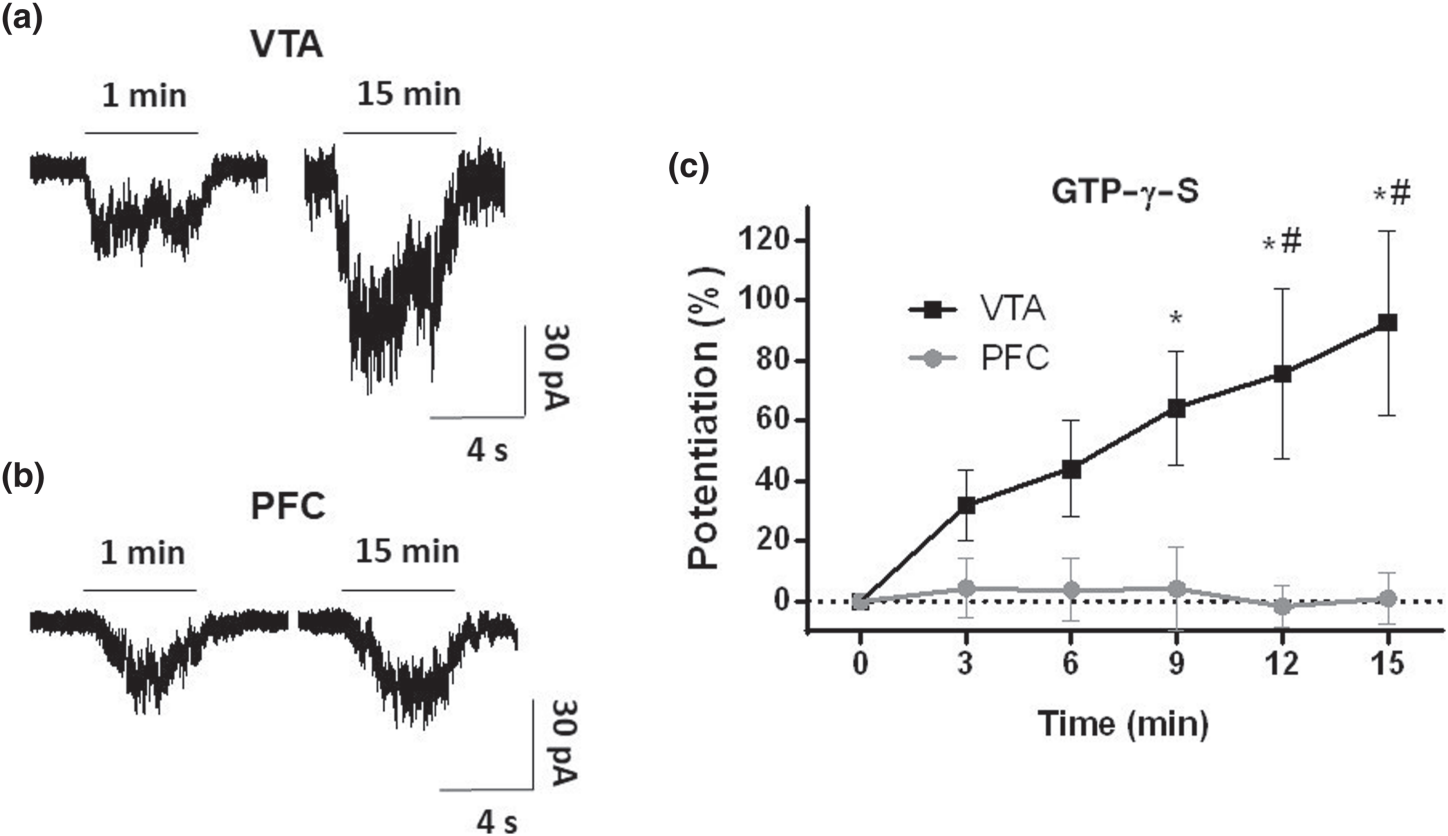
Effects of intracellular GTP‐γ‐S on glycine receptors in VTA and PFC in WT mice. (a, b) Currents in activated glycine receptors were assayed using 15 and 30 μM glycine every 3 min for VTA and PFC neurons, respectively, with intracellular dialysis of GTP‐γ‐S. Traces show only the first and last evoked currents (at 1 and 15 min). (c) Time course of GTP‐γ‐S mediated potentiation of glycine receptors in VTA and PFC. Data represented as mean ± SEM; *n* = 11 neurons for VTA and *n* = 10 neurons for PFC. ^*^ *P* < 0.05, VTA significantly different from PFC; two‐way ANOVA and Bonferroni post hoc test. ^#^ *P* < 0.05, significantly different from the control in the VTA curve; one‐way ANOVA

It was previously reported that glycine receptors containing α1 subunits are potentiated by ethanol and Gβγ and that this effect was not seen in glycine receptors containing α2 or α3 (Sánchez et al., 2015; Yevenes et al., 2010). The positive allosteric modulation of α1 glycine receptors appears to result from an interaction between basic amino acids in the intracellular loop of the α1 subunit with the Gβγ complex available from the dissociation of the trimeric G protein (Yevenes et al., 2008; Zhu & Ye, 2005). We tested the effect of GTP‐γ‐S, a nonhydrolysable GTP analogue (Yevenes et al., 2003), on the glycine‐activated current in order to evaluate the presence of the α1 subunit in the VTA and PFC. Activation of G proteins with intracellular GTP‐γ‐S applied via the recording pipette for 15 min increased the amplitude of the Cl^−^ current as previously reported for glycine receptors in the spinal cord and nAc (Mariqueo et al., 2014; Muñoz et al., 2019). In this study, dialysis of GTP‐γ‐S potentiated the currents evoked by 15 μM glycine in neurons of the VTA (Figure 5a). Under this condition, the glycine current amplitude of VTA neurons almost doubled after 15 min of GTP‐γ‐S dialysis. Using an equipotent glycine concentration of 30 μM in the PFC, no effect was detected (Figure 5b). The curves in Figure 5c show the potentiation of the glycine current only in the VTA. The VTA potentiation is similar to that previously reported for recombinant α1 glycine receptors by Gβγ (Yevenes et al., 2003). On the other hand, the absence of a potentiating effect in the PFC suggests that α2 and α3 subunits were expressed more than α1 subunits in PFC neurons.

### 3.6 Sensitivity of glycine receptors to ethanol in the VTA and PFC

**FIGURE 6:**
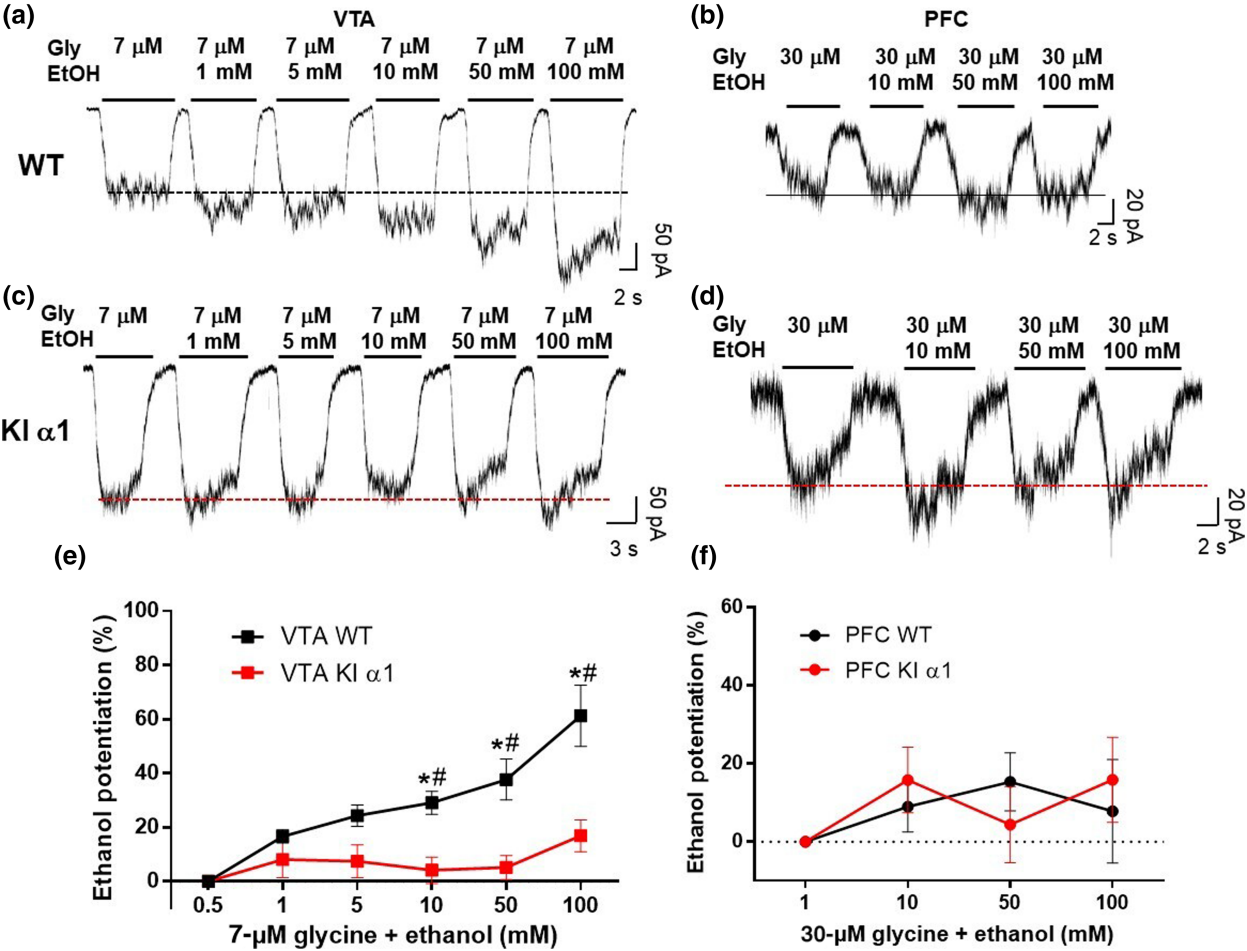
Effects of ethanol on glycine receptors of VTA and PFC from WT and α1 KI mice. (a, c) Representative traces of glycine‐activated currents and the effects of 1 to 100 mM ethanol in neurons of the VTA from WT and α1 KI mice, respectively. (b, d) Representative traces of glycine‐activated currents and the effects of 10 to 100 mM ethanol in neurons of the PFC from WT and α1 KI mice, respectively. The currents were activated with 7 and 30 μM glycine for VTA and PFC, respectively. (e) Graph shows ethanol potentiation (1–100 mM) of glycine receptors of VTA from WT and α1 KI mice. One outlier was found and excluded from this graph. (f) Graph shows ethanol potentiation (10–100 mM) of glycine receptors in PFC from WT and α1 KI mice. Data presented are means ± SEM; *n* = 10 for 1 mM, *n* = 10 for 5 mM, *n* = 16 for 10 mM, *n* = 15 for 50 mM and *n* = 14 for 100 mM in VTA of WT mice; *n* = 7 for 1 mM, *n* = 7 for 5 mM, *n* = 8 for 10 mM, *n* = 8 for 50 mM and *n* = 8 for 100 mM in VTA of α1 KI mice. *n* = 6 for 10 mM, *n* = 6 for 50 mM and *n* = 6 for 100 mM in PFC of WT mice; *n* = 14 for 10 mM, *n* = 14 for 50 mM and *n* = 14 for 100 mM in PFC α1 KI mice. ^*^ *P* < 0.05, significant effects of the different ethanol concentrations between the different potentiation curves from different brain regions; two‐way ANOVA and Bonferroni post hoc test. ^#^ *P* < 0.05, significant effects of the different ethanol concentrations within the same potentiation curve of the same brain region; one‐way ANOVA

It has been reported that the glycine‐activated Cl^−^ currents in the spinal cord (Aguayo et al., 2014), hypoglossal motoneurons (Eggers & Berger, 2004) and the nAc (Gallegos et al., 2019) are sensitive to low concentrations of ethanol. To further study the pharmacological sensitivity of glycine receptors in the VTA and PFC, we examined the effects of ethanol (1–100 mM) on the amplitude of glycine currents in acutely dissociated neurons from WT mice. In neurons of the nAc, although synaptic glycinergic currents were unchanged by ethanol, the tonic glycinergic currents and currents activated by low concentrations of exogenous glycine were potentiated by ethanol (Gallegos et al., 2019). Therefore, in the present study, we examined currents activated by an EC_10–20_ of glycine in the presence of ethanol. In WT mice, the glycine receptors in VTA neurons were highly sensitive to low concentrations of ethanol (1 and 5 mM), as well as being potentiated by the higher concentrations (Figure 6a,e). On the other hand, minimal potentiation by ethanol was found in the PFC, with 10, 50 and 100 mM ethanol (Figure 6b,f). To further evaluate the contribution of the α1 subunit in the glycine receptors of these regions, we used a genetically modified mouse, the α1 KI mouse (Figure 6c), which has a mutation that largely attenuates ethanol potentiation (Aguayo et al., 2014; Muñoz et al., 2019). The results found with the α1 KI mouse showed that the effect of ethanol was very significantly attenuated in neurons of the VTA (Figure 6e), confirming that α1 subunits were present in the VTA. In the PFC, the data were similar to WT neurons because ethanol did not significantly alter the glycine current amplitude (Figure 6f). In agreement with previous results in this study, the findings with ethanol suggest an important contribution of the α1 subunit to the total ethanol potentiation in the VTA, but not in the PFC. Figure S2 shows the glycine concentration–response curve in VTA neurons (EC_50_ = 34 ± 4 μM) and PFC (EC_50_ = 90 ± 9) from the α1 KI mice.

### 3.7 Further evidence that glycine receptors in the VTA are primarily α1‐containing receptors

The previous results suggested that the glycine receptors in the VTA contain α1 subunits. On the other hand, the PFC appears to express α2 or α3 subunits that are insensitive to ethanol and GTP‐γ‐S. To study the possible contribution of glycine receptors containing α2 subunits in the VTA and PFC, we examined glycine‐activated currents in neurons derived from the α2 KO mouse (Avila, Vidal, et al., 2013). Recordings performed in VTA neurons showed that these neurons still exhibited glycine‐activated currents (Figure S3) and the concentration–response curve to glycine in the VTA was shifted to the right when compared with WT neurons (Figure S3C), with an EC_50_ value of 56 ± 11 μM. Preliminarily, the EC_50_ in PFC neurons from the α2 KO mice was 87 ± 15 μM and also displaced to the right, when compared with data from WT mice. The data also show that the glycine current density was reduced in both brain regions compared with WT mice (Figure S3D). Interestingly, although glycine was able to elicit glycine‐activated currents in all the VTA neurons examined, only small‐amplitude currents in four out of nine neurons were recorded in the PFC. These results suggest differences in the glycine receptor subunits expressed in both regions and a major contribution of α2 subunits to the glycine receptors in the PFC.

To further confirm that the α2 subunit is part of the glycine receptor complex in these brain regions, we performed Western blot analysis and found that the expression of the α subunits (pan α antibody) was reduced in the VTA and PFC in α2 KO mice as compared with WT mice (Figure S4A). As expected, the reduction was more evident in the PFC than in the VTA (Figure S4B), suggesting that the α2 subunit is present in both of these regions, but with much higher levels in the PFC where the total α subunit levels were reduced fourfold (Figure S4). As expected, the expression of the β subunit was not affected in the α2 KO mice, and the β band was similar in the VTA and PFC from WT and α2 KO mice (Figure S4C,D).

**FIGURE 7:**
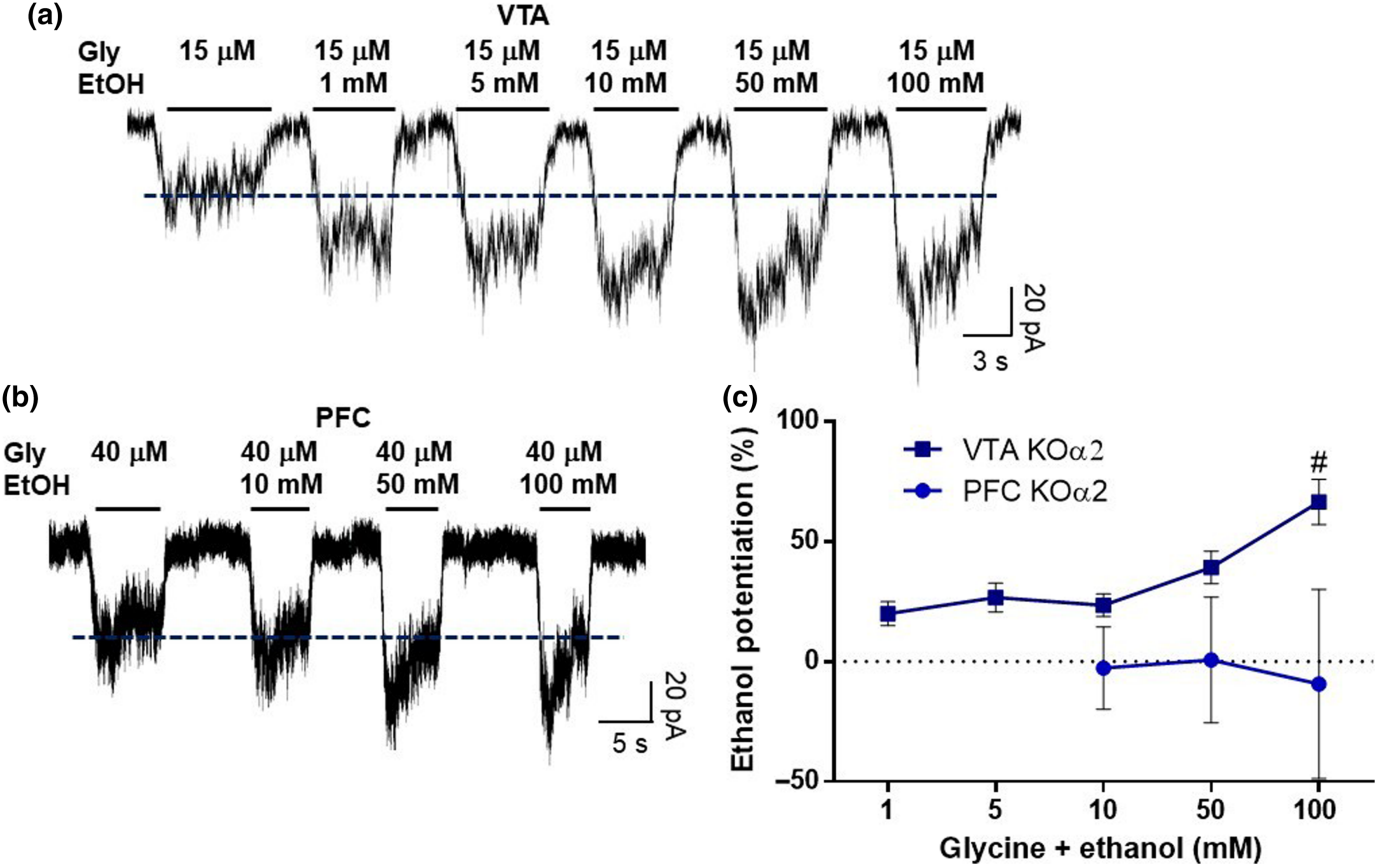
Effects of ethanol on glycine receptors of VTA and PFC from α2 KO mice. (a) Representative traces of currents from VTA neurons evoked with 15 μM of glycine and the effect of 1–100 mM of ethanol. (b) Representative traces of PFC neurons evoked with 40 μM of glycine and the effect of 10–100 mM of ethanol. (c) Graph shows the effect of several concentrations of ethanol on the amplitude of glycine‐activated currents for VTA and PFC of α2 KO mice. Data presented are means ± SEM; *n* = 9 for 1 mM, *n* = 9 for 5 mM, *n* = 21 for 10 mM, *n* = 20 for 50 mM and *n* = 18 for 100 mM of VTA. *n* = 3 for 10 mM, *n* = 3 for 50 mM and *n* = 3 for 100 mM of PFC. ^#^ *P* < 0.05, significant effects of the different ethanol concentrations in VTA; one‐way ANOVA

Finally, to further examine the role of α subunits on the ethanol potentiation of VTA and PFC neurons, we characterized the effects of ethanol (1–100 mM) on the glycine current amplitude in VTA and PFC neurons from the α2 KO mice (Figure 7a,c). The results showed that glycine receptors in the VTA from α2 KO neurons were still positively modulated by ethanol. In KO mice, only a few PFC neurons showed sizable glycine‐activated currents (three out of 21) precluding further analysis. These three PFC neurons were insensitive to ethanol (10, 50 and 100 mM) (Figure 7b,c). The glycine EC_50_ values and the degree of ethanol potentiation in the VTA and PFC in the three mouse models used in this study are summarized in Table 1.

**TABLE 1:** Summary of the properties found in glycine receptors of VTA and PFC from WT, α1 KI and α2 KO mice

Mouse model	VTA			PFC		
	WT	α1 KI	α2 KO	WT	α1 KI	α2 KO
Glycine EC_50_ (μM)	35 ± 5 (*n* = 16)	34 ± 4 (n.s.) (*n* = 14)	56 ± 11* (*n* = 9)	75 ± 8 (*n* = 11)	90 ± 9 (n.s.) (*n* = 11)	87 ± 15 (n.s.) (*n* = 4)
Potentiation 10 mM ethanol (%)	36 ± 8 (*n* = 16)	4 ± 5* (*n* = 8)	28 ± 4 (n.s.) (*n* = 21)	7 ± 5 (*n* = 8)	16 ± 8 (n.s.) (*n* = 14)	−3 ± 17 (n.s.) (*n* = 3)
Potentiation 100 mM ethanol (%)	78 ± 19 (*n* = 14)	17 ± 6* (*n* = 8)	69 ± 8 (n.s.) (*n* = 18)	1 ± 9 (*n* = 8)	16 ± 11 (n.s.) (*n* = 14)	−9 ± 39 (n.s.) (*n* = 3)

*Note:* All values shown are means ± SEM. The table shows the values for EC_50_ obtained in the VTA and PFC for the three different mouse models studied. The EC_50_s in α1 KI and α2 KO were compared with WT using one‐way ANOVA and Bonferroni post hoc test. Ethanol potentiation corresponds to the change in amplitude between control with glycine EC_10–20_ (equipotent) and in the presence of 10 or 100 mM ethanol. Percentages of potentiation in α1 KI or α2 KO were compared with WT using one‐way ANOVA and Bonferroni post hoc test.

Abbreviations: *n*, number of cells; n.s., not significant *P* > 0.05; PFC, prefrontal cortex; VTA, ventral tegmental area; WT, wild‐type; α1 KI, glycine receptor α1 knock‐in; α2 KO, glycine receptor α2 knockout.

* *P* < 0.05, significantly different from WT.

## 4 DISCUSSION

### 4.1 Strychnine‐sensitive glycine receptors in supraspinal CNS regions

The presence of functional glycine receptors in several higher brain regions such as the hippocampus (Xu & Gong, 2010; Zhang et al., 2008), dorsal raphe (Maguire et al., 2014), cerebellum (Richardson & Rossi, 2017), striatum (Molchanova et al., 2018) and areas of the reward system is an emerging area of study. The modulation of glycine receptors by ethanol has been reported in the mouse nAc (Gallegos et al., 2019) and the rat VTA (Ye et al., 2001). In the present study, we characterized the presence of glycine receptors in slices and dissociated neurons from the VTA and PFC using electrophysiology in three mouse models. Additionally, using the GlyT2‐GFP mouse line, we demonstrated the presence of glycinergic axons and the apposition of glycine receptor and GlyT2 IHC signals in the VTA and PFC. This was further confirmed by recording strychnine‐sensitive glycinergic synaptic transmission in these regions after pharmacological blockade of other ligand‐gated ion channels such as the AMPA, NMDA, GABA_A_ and nACh receptors (Figure 2). We also found a greater proportion of GlyT2‐GFP‐positive fibres in VTA consistent with a higher number of neurons that presented glycinergic synaptic events in VTA compared with PFC. Lastly, the glycine‐mediated currents in VTA and PFC had similar characteristics to glycinergic currents reported in the nAc (Muñoz et al., 2018).

### 4.2 Glycine receptor subunits can be differentiated by their electrophysiological and pharmacological properties

Although IHC and Western blots confirmed the presence of glycine receptors in VTA and PFC neurons, these results did not allow us to conclusively identify specific α subunits, which is why we used patch‐clamp techniques to evaluate the presence of functional glycine receptors in these brain areas. The analysis of synaptic activity in the VTA and PFC suggests the presence of different glycine receptor isoforms because of the difference in decay constant values obtained that are in agreement with those reported in cultured spinal cord neurons and HEK cells (Mariqueo et al., 2014; Raltschev et al., 2016; Zhang et al., 2015). Also, previous studies in HEK cells showed EC_50_ values of ~45 and 66 μM for glycine receptors containing α1 and α2 subunits, respectively, whereas glycine receptors containing the α3 subunit showed values of 90 μM or higher (Miller et al., 2004; Sánchez et al., 2015). Currents in the VTA and PFC differed in their sensitivity to glycine with EC_50_ values of 30 ± 2 and 70 ± 7 μM, respectively (Figure 3). The results agree with those reported in rat VTA neurons that exhibited an EC_50_ between 32 and 37 μM (Ye, 2000). It is well accepted that glycine receptor α1 subunits have a higher sensitivity to glycine than α2 and α3 subunits (Sánchez et al., 2015; Yevenes & Zeilhofer, 2011b). Thus, VTA appears to express α1 glycine receptors, whereas PFC is likely to express α2 and/or α3 subunits. In dissociated rat PFC neurons (6–39 postnatal), glycine‐evoked currents had EC_50_ values from 58 to 117 μM (Lu & Ye, 2011). In the present study, PFC neurons showed an EC_50_ of 70 ± 7 μM. This value is different from that reported in another study using brain slices (McCracken et al., 2017), which showed that neurons perfused with glycine elicited a slow rising Cl^−^ current with an EC_50_ of 674 ± 32 μM. The large difference in receptor sensitivity with our study may be related to their application methodology that is likely confounded by dilution and reuptake of the neurotransmitter in the slice.

In addition, the α1, but not α2 or α3 subunits, are modulated positively by Gβγ (Burgos et al., 2015; Yevenes et al., 2010). Therefore, we concluded that if the glycine‐activated Cl^−^ current in VTA was potentiated by GTP‐γ‐S, the receptor complex contains α1 subunits. As shown in Figure 5, the glycine receptors in VTA neurons were significantly potentiated in the presence of GTP‐γ‐S. At 15 min after dialysis, the percentage of potentiation of the glycine current was 93 ± 30%. A different result was found in the PFC where the application of GTP‐γ‐S was unable to modulate the current. Thus, the present results support a predominant presence of the α1 subunit in the VTA, but not in the PFC. Although phosphorylation and receptor density can affect some physiological characteristics of glycine receptors (Moraga‐Cid et al., 2020; Taleb & Betz, 1994) and might explain some of the distinct properties found in VTA and PFC neurons, we believe that these differences are related to differences on subunit expression as discussed previously.

This analysis is further supported by the sensitivity of glycine receptors to ethanol in the α1 KI and α2 KO mice, summarized in Table 1. Wild‐type VTA neurons displayed high sensitivity to ethanol with substantial potentiation at 10, 50 and 100 mM, but not in the α1 KI mouse where the potentiation was virtually abolished, and this in agreement with previous results from α1 KI spinal cord neurons (Aguayo et al., 2014). The results obtained in the α2 KO mice confirmed the idea that VTA mainly expresses α1 subunits because the sensitivity to ethanol in these neurons was not significantly affected and every neuron recorded presented glycine‐activated currents. An interesting finding in VTA neurons of the α2 KO mouse was the shift in EC_50_ values from 30 ± 2 to 56 ± 11 μM and the reduction of the glycine current density. This change suggests that the α2 subunit is important in this region in native conditions. Although α2 splicing variants with different glycine sensitivity have been described (Miller et al., 2004), and unpublished data from our laboratory also showed that the α2β subunit conformation of glycine receptors is highly sensitive to ethanol, the α2 subunits are totally absent from the α2 KO mice. Thus, a more likely explanation for this change could be compensatory processes occurring in the KO model, as recently published (San Martin et al., 2020), by other glycine receptor α subunits (e.g., α3 and α4). In conclusion, glycine receptor α2 subunits might be expressed in VTA, but only a small proportion.

The glycine receptors in PFC neurons were insensitive to ethanol in all three mouse models examined. The lack of potentiation in WT neurons ruled out the contribution of α1 subunits. Interestingly, in the α2 KO model, in the few PFC neurons that showed glycine‐activated currents, current density was reduced, but not completely abolished. We were able to perform a dose–response curve in a few neurons that presented a maximal amplitude above ~130 pA at a saturating glycine concentration. Hence, the glycine receptors in the PFC are likely to be composed of α2/α3 subunits.

Finally, the presence of glycine receptor β subunits in Western blot assays suggests the presence of both homomeric and heteromeric glycine receptor complexes in these two regions. This is supported by data that showed that glycine‐activated currents in VTA and PFC neurons were partly inhibited by picrotoxin (Figure 4). This channel blocker inhibits homomeric glycine receptors by more than 70%, leaving the heteromeric recptors largely unaffected (Lu & Ye, 2011; Lynch, 2009; Maleeva et al., 2017). In the PFC and VTA, the inhibition was 40 ± 5% and 50 ± 4%, respectively, suggesting the presence of heteromeric glycine receptors. The heterogeneity in terms of picrotoxin sensitivity in the VTA could be due to the heterogeneous neuronal population reported in the VTA (Morales & Margolis, 2017); however, the majority of the neurons recorded support the presence of heteromeric receptors, partly inhibited by picrotoxin.

### 4.3 Role of glycine receptors in the mesolimbic system

The mesolimbic reward system is critical for the rewarding properties of natural stimuli and drugs of abuse. The glycine receptors in the nAc have been recently shown to regulate nAc neuron excitability in the presence of ethanol (Gallegos et al., 2019; Muñoz et al., 2019). In this study, we provide evidence for the existence of an additional inhibitory component in two other regions of the mesolimbic circuit that are connected to the nAc. This could be relevant because studies have shown that the animals used in this work exhibited higher ethanol consumption than the WT mice (Muñoz et al., 2019; San Martin et al., 2020).

The presence of glycine receptors in VTA neurons is interesting for two reasons. First, the high sensitivity of these receptors to glycine in this region, where a low concentration of glycine (5–10 μM) was able to activate a large‐amplitude current, suggests that they might be active at a physiological glycine concentration in the CSF (Stover et al., 1997). Evidence of a tonic activation of glycine receptors in VTA neurons (Wang et al., 2005; Ye et al., 2004) suggests that glycine receptors play a role in the basal control of the mesolimbic pathway and thus rewarding and addictive behaviours. Second, because these glycine receptors are highly sensitive to ethanol, the excitability of VTA neurons is expected to be reduced, similar to the nAc (Gallegos et al., 2019; Muñoz et al., 2019). This is interesting because ethanol can affect dopamine release in the nAc (Bassareo et al., 2017; Molander & Söderpalm, 2005) and because the soma of these dopaminergic neurons are in the VTA, it could be an additional mechanism contributing to the addictive effects of ethanol.

In conclusion, our study indicates that neurons in two mesolimbic regions, the VTA and the PFC, express glycine receptors with different functional properties that are associated with distinct subunit compositions. VTA neurons express predominantly ethanol‐sensitive α1‐containing glycine receptors that may have implications for the addictive properties of ethanol. By contrast, studies with the glycine receptor α1 KI and α2 KO mice suggest that ethanol‐insensitive glycine receptors in the PFC contain predominantly α2/α3 subunits.

## AUTHOR CONTRIBUTIONS

A.A., S.G., R.V., L.S.M., L.G.A. and B.M. planned and performed the experiments, collected and analysed the data, and prepared all the figures. A.A., S.G. and L.G.A. wrote the manuscript. R.J.H. and H.U.Z. contributed to the writing of the manuscript and provided breeding pairs of α2 KO and GlyT2‐GFP mice. All authors read and approved the final version of the manuscript.

## CONFLICT OF INTEREST

The authors declare no conflicts of interest.

## DECLARATION OF TRANSPARENCY AND SCIENTIFIC RIGOUR

This Declaration acknowledges that this paper adheres to the principles for transparent reporting and scientific rigour of preclinical research as stated in the *BJP* guidelines for Design & Analysis, Immunoblotting and Immunochemistry, and Animal Experimentation and as recommended by funding agencies, publishers and other organizations engaged with supporting research.

## Supporting information


**Figure S1.** Brain slices containing the VTA or PFC. Schematic representations of coronal brain slices containing the VTA (**A**) and PFC (**B**) that show the region where neurons were selected for electrophysiology experiments and to microdissect the area for neuronal dissociation. Image credit: Allen Institute. ©2004 Allen Institute for Brain Science. Allen Mouse Brain Atlas. Available from: atlas.brain-map.org/atlas.
**Figure S2.** Glycine‐activated currents in dissociated neurons from the VTA and PFC of α1 KI mice. **A)** Representative traces for glycine‐activated currents with 1–1000 μM glycine in VTA neurons. **B)** Representative traces for glycine‐activated currents with 10–1000 μM glycine in PFC neurons. **C)** Graph shows glycine concentration‐response curves normalized to the maximum response (100%) for neurons in VTA (dark red squares) and PFC (dark circles). Data represented as mean ± SEM, n = 14 for VTA and n = 11 for PFC.
**Figure S3.** Glycine‐activated currents in dissociated neurons from the VTA and PFC of α2 KO mice. **A)** Representative traces for glycine‐activated currents with 1–1000 μM glycine in VTA neurons. **B)** Representative traces for glycine‐activated currents with 10–1000 μM glycine in PFC neurons. **C)** Graph shows glycine concentration‐response curves normalized to the maximum response (100%) for neurons in the VTA (dark blue squares) and PFC (blue circles) (n = 9 for VTA and n = 4 for PFC). **D)** Scatter graph shows glycine current density in VTA and PFC of WT and α2 KO mice (VTA WT n = 45, VTA α2 KO n = 28, PFC WT n = 21, PFC α2 KO n = 31). Data represented as mean ± SEM, Mann–Whitney test for D, *p < 0.05.
**Figure S4.** Presence of the α and β GlyR subunits in the VTA and PFC of WT and α2 KO mice. **A,B)** Western blot and quantification of pan α GlyR subunits in the VTA and PFC of WT and α2 KO mice, respectively. The signal was normalized to the expression of Gβ. **C,D)** Western blot and quantification of β GlyR subunits in the VTA and PFC of WT and α2 KO mice, respectively. The signal was normalized to the expression of Gβ. Data represented as mean ± SEM, n = 4 for pan α GlyR and n = 3 for β GlyR of VTA WT and for α2 KO. n = 3 for pan α GlyR and n = 4 for β GlyR of PFC WT and for α2 KO mice.

## Data Availability

The data that support the findings of this study are available from the corresponding author upon reasonable request. Some data may not be made available because of privacy or ethical restrictions.
