# Supplementary material for: Presence of ethanol‐sensitive and ethanol‐insensitive glycine receptors in the ventral tegmental area and prefrontal cortex in mice
Source: Br J Pharmacol. 2021 Sep 17;178(23):4691–707. doi: 10.1111/bph.15649 (PMC9293192; doi:10.1111/bph.15649)
Supplement: Supplementary file 1 — Figure S1. Brain slices containing the VTA or PFC. Schematic representations of coronal brain slices containing the VTA (A) and PFC (B) that show the region where neurons were selected for electrophysiology experiments and to microdissect the area for neuronal dissociation. Image credit: Allen Institute. ©2004 Allen Institute for Brain Science. Allen Mouse Brain Atlas. Available from: atlas.brain-map.org/atlas. Figure S2. Glycine‐activated currents in dissociated neurons from the VTA and PFC of α1 KI mice. A) Representative traces for glycine‐activated currents with 1–1000 μM glycine in VTA neurons. B) Representative traces for glycine‐activated currents with 10–1000 μM glycine in PFC neurons. C) Graph shows glycine concentration‐response curves normalized to the maximum response (100%) for neurons in VTA (dark red squares) and PFC (dark circles). Data represented as mean ± SEM, n = 14 for VTA and n = 11 for PFC. Figure S3. Glycine‐activated currents in dissociated neurons from the VTA and PFC of α2 KO mice. A) Representative traces for glycine‐activated currents with 1–1000 μM glycine in VTA neurons. B) Representative traces for glycine‐activated currents with 10–1000 μM glycine in PFC neurons. C) Graph shows glycine concentration‐response curves normalized to the maximum response (100%) for neurons in the VTA (dark blue squares) and PFC (blue circles) (n = 9 for VTA and n = 4 for PFC). D) Scatter graph shows glycine current density in VTA and PFC of WT and α2 KO mice (VTA WT n = 45, VTA α2 KO n = 28, PFC WT n = 21, PFC α2 KO n = 31). Data represented as mean ± SEM, Mann–Whitney test for D, *p < 0.05. Figure S4. Presence of the α and β GlyR subunits in the VTA and PFC of WT and α2 KO mice. A,B) Western blot and quantification of pan α GlyR subunits in the VTA and PFC of WT and α2 KO mice, respectively. The signal was normalized to the expression of Gβ. C,D) Western blot and quantification of β GlyR subunits in the VTA and PFC of WT and α2 KO mice, respectively. The [file BPH-178-4691-s001.docx]

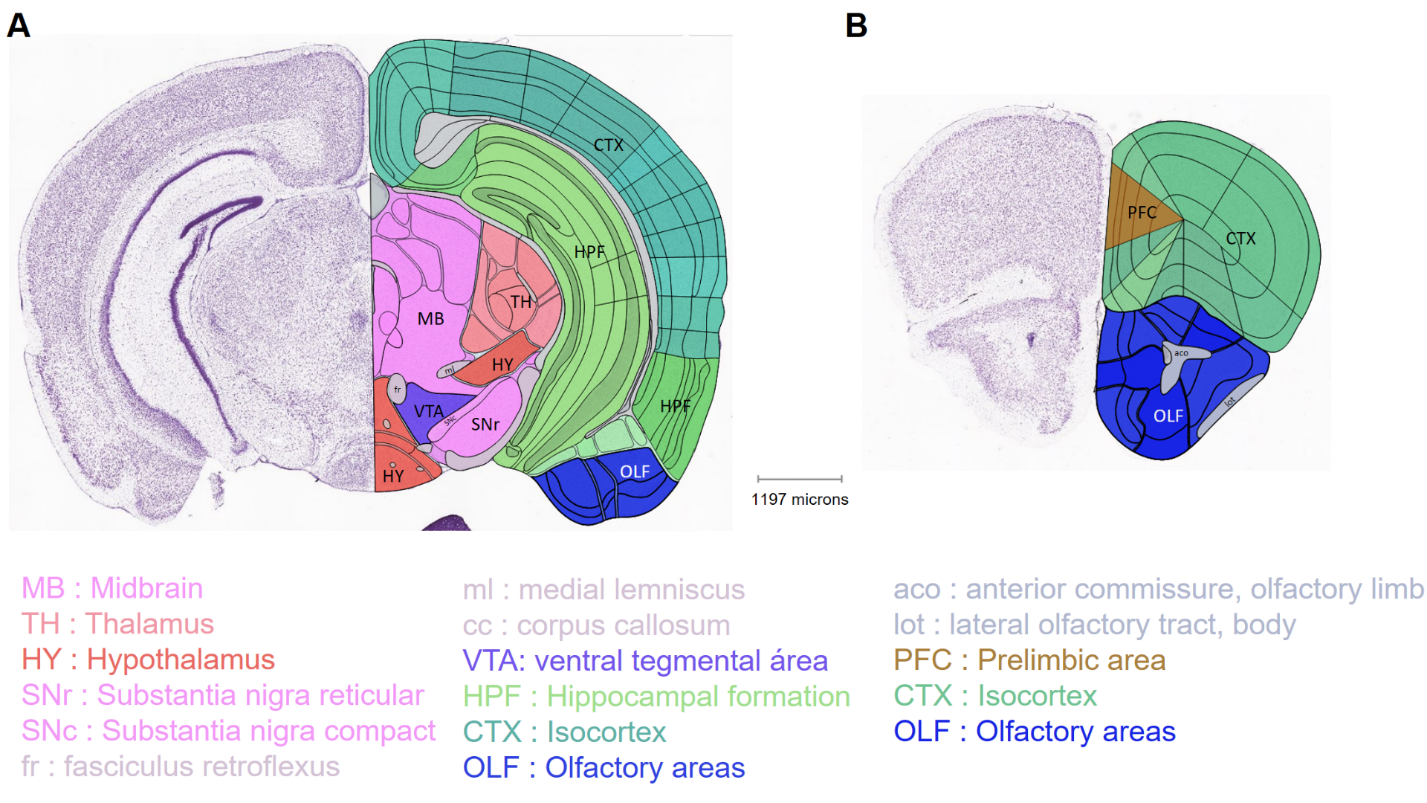
**Supplementary figure 1**

**Supplementary figure 1.** Brain slices containing the VTA or PFC. Schematic representations of coronal brain slices containing the VTA (**A**) and PFC (**B**) that show the region where neurons were selected for electrophysiology experiments and to microdissect the area for neuronal dissociation. Image credit: Allen Institute. ©2004 Allen Institute for Brain Science. Allen Mouse Brain Atlas. Available from: atlas.brain-map.org/atlas**.**


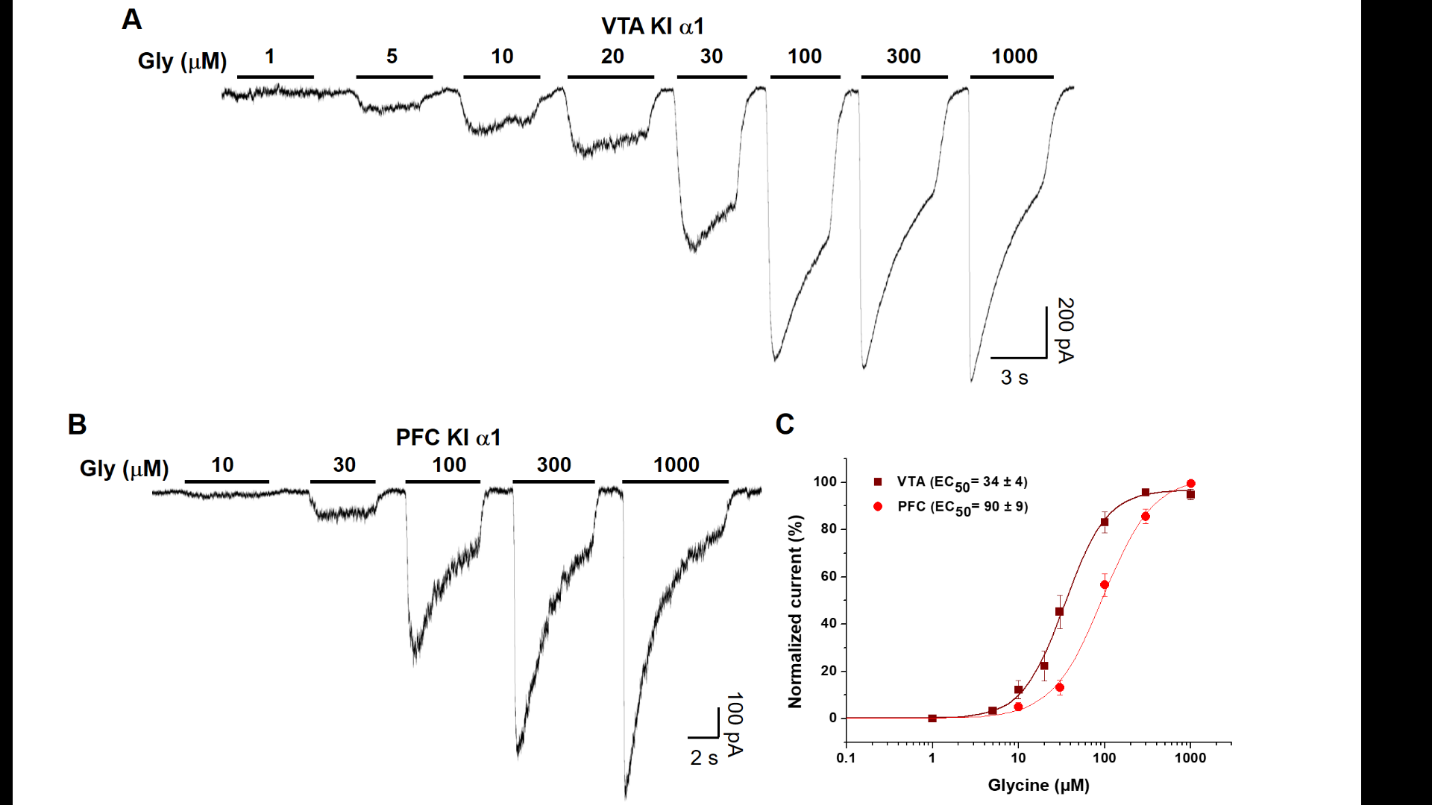
**Supplementary figure 2**

**Supplementary figure 2.** Glycine-activated currents in dissociated neurons from the VTA and PFC of α1 KI mice. **A)** Representative traces for glycine-activated currents with 1-1000 μM glycine in VTA neurons. **B)** Representative traces for glycine-activated currents with 10-1000 μM glycine in PFC neurons. **C)** Graph shows glycine concentration-response curves normalized to the maximum response (100%) for neurons in VTA (dark red squares) and PFC (dark circles). Data represented as mean ± SEM, n=14 for VTA and n=11 for PFC.


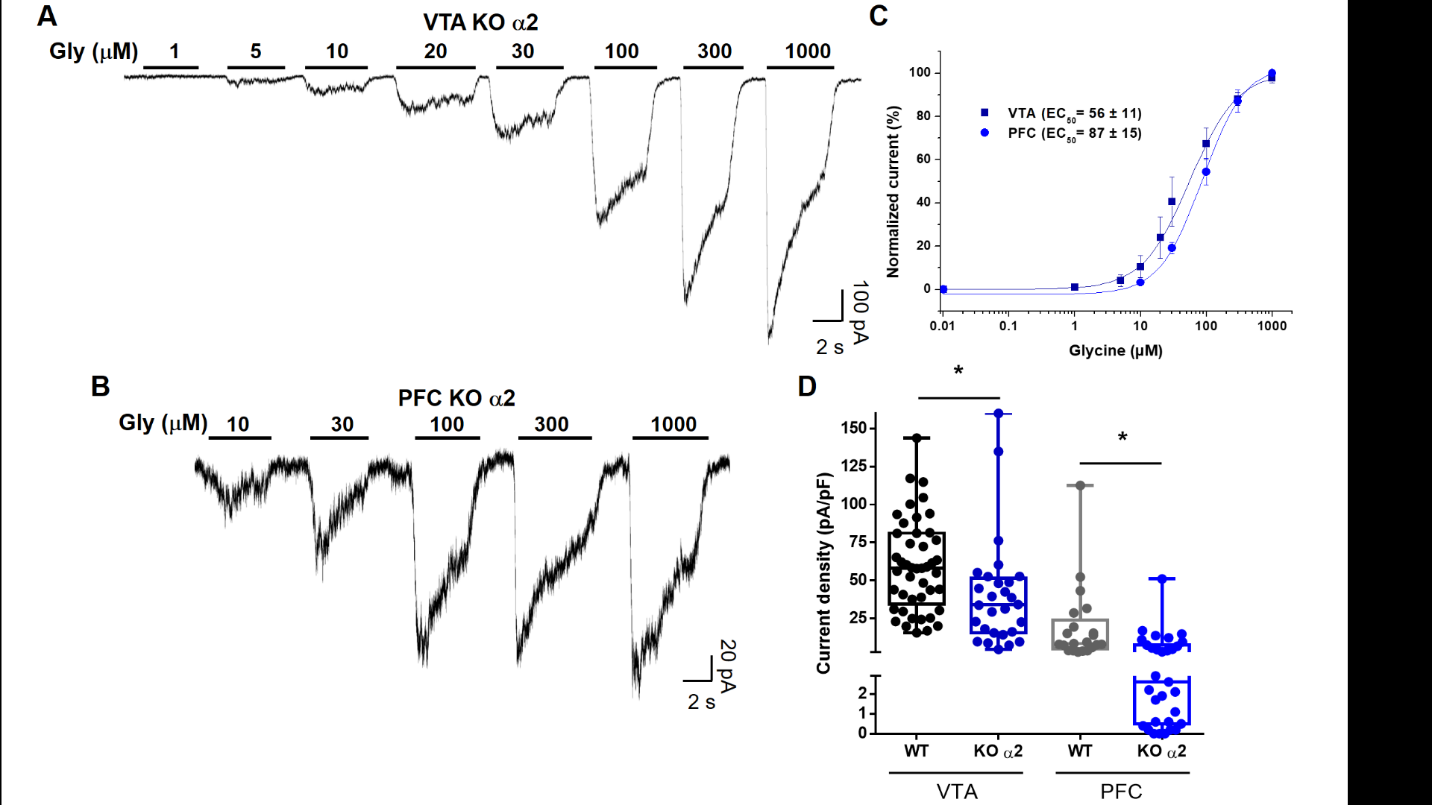
**Supplementary figure 3**.

**Supplementary figure 3**. Glycine-activated currents in dissociated neurons from the VTA and PFC of α2 KO mice. **A)** Representative traces for glycine-activated currents with 1-1000 μM glycine in VTA neurons. **B)** Representative traces for glycine-activated currents with 10-1000 μM glycine in PFC neurons. **C)** Graph shows glycine concentration-response curves normalized to the maximum response (100%) for neurons in the VTA (dark blue squares) and PFC (blue circles) (n=9 for VTA and n=4 for PFC). **D)** Scatter graph shows glycine current density in VTA and PFC of WT and α2 KO mice (VTA WT n=45, VTA α2 KO n=28, PFC WT n=21, PFC α2 KO n=31). Data represented as mean ± SEM, Mann-Whitney test for D, *p< 0.05.


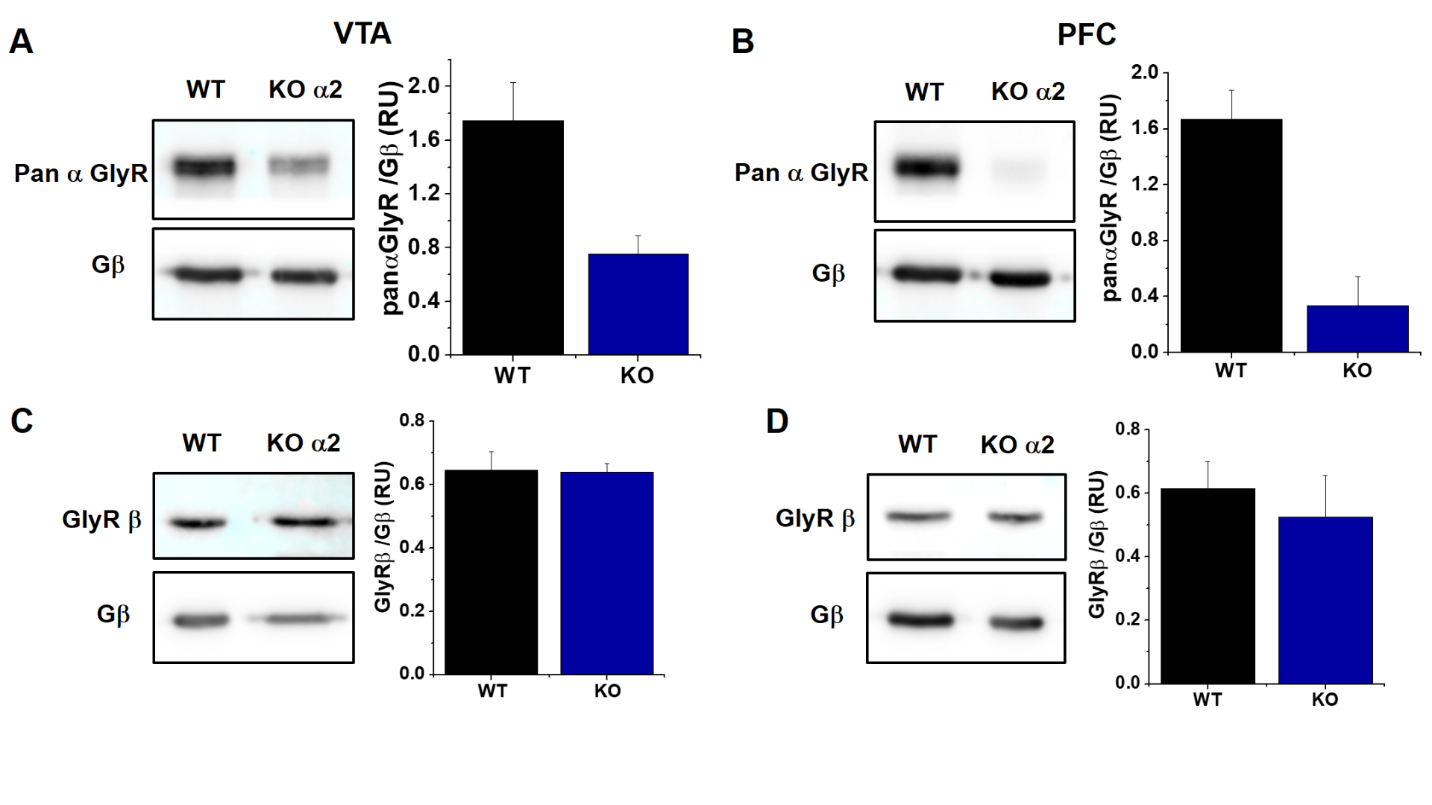
**Supplementary figure 4**.

**Supplementary figure 4**. Presence of the α and β GlyR subunits in the VTA and PFC of WT and α2 KO mice. **A,B)** Western blot and quantification of pan α GlyR subunits in the VTA and PFC of WT and α2 KO mice, respectively. The signal was normalized to the expression of Gβ. **C,D)** Western blot and quantification of β GlyR subunits in the VTA and PFC of WT and α2 KO mice, respectively. The signal was normalized to the expression of Gβ. Data represented as mean ± SEM, n=4 for pan α GlyR and n=3 for β GlyR of VTA WT and for α2 KO. n=3 for pan α GlyR and n=4 for β GlyR of PFC WT and for α2 KO mice.
